# Current Status and Future Directions of Immunotherapies in Soft Tissue Sarcomas

**DOI:** 10.3390/biomedicines10030573

**Published:** 2022-02-28

**Authors:** William G. J. Kerrison, Alexander T. J. Lee, Khin Thway, Robin L. Jones, Paul H. Huang

**Affiliations:** 1 Division of Molecular Pathology, The Institute of Cancer Research, Sutton SM2 5NG, UK; will.kerrison@icr.ac.uk (W.G.J.K.); khin.thway@rmh.nhs.uk (K.T.); 2 The Christie NHS Foundation Trust, Manchester M20 4BX, UK; alexander.lee1@nhs.net; 3 The Royal Marsden NHS Foundation Trust, London SW3 6JJ, UK; robin.jones4@nhs.net; 4 Division of Clinical Studies, The Institute of Cancer Research, London SW3 6JB, UK

**Keywords:** sarcoma, immunotherapy, tissue sarcoma

## Abstract

Immunotherapy in soft tissue sarcoma (STS) has experienced a surge of interest in the past decade, contributing to an expanding number of therapeutic options for this extremely heterogenous group of rare malignancies. Immune checkpoint inhibitors (CPIs) targeting the PD-1 and CTLA-4 axes have demonstrated promising responses in a select number of STS subtypes, including rarer subtypes, such as alveolar soft part sarcoma, SWI/SNF-deficient sarcomas, clear cell sarcoma, and angiosarcoma. Multiple pan-subtype sarcoma trials have facilitated the study of possible predictive biomarkers of the CPI response. It has also become apparent that certain therapies, when combined with CPIs, can enhance response rates, although the specific mechanisms of this possible synergy remain unconfirmed in STS. In addition to CPIs, several other immune targeting agents, including anti-tumour-associated macrophage and antigen-directed therapies, are now under assessment in STS with promising efficacy in some subtypes. In this article, we review the state of the art in immunotherapy in STS, highlighting the pre-clinical and clinical data available for this promising therapeutic strategy.

## 1. Introduction

Soft tissue sarcomas (STS) are a group of over 100 rare cancers of definite or suspected mesenchymal origin/differentiation, typically classified by morphological and immunophenotypic characteristics. Such a classification, however, only partly describes the wide range of clinical behaviour that is exhibited both within and between the histologically defined subtypes. Encouragingly, the understanding of the disparate tumour biology that underpins this clinical heterogeneity has increased enormously over the past two decades [1,2]. A significant proportion of STS are now recognised to possess highly recurrent, subtype-specific genetic aberrations that play central roles in tumorigenesis via such processes as epigenetic reprogramming or upregulation of oncogenic pathway signalling. Meanwhile, other STS subtypes are known to be associated with a high degree of chromosomal copy number abnormalities. However, despite this improved knowledge, progress in developing effective new systemic therapies in STS has been disappointingly slow. For a majority of patients with STS, who develop unresectable distant disease, palliative treatment options remain limited to the same narrow repertoire of frequently ineffective, primarily cytotoxic, drugs [3]. There remains an urgent need for effective new drug therapies for these diseases.

Over the past decade, the development of novel therapies that mobilise the body’s immune system in attacking tumours has revolutionised the treatment of a still-growing list of solid and haematological malignancies. However, the current era of immuno-oncology (IO) has yet to have a major impact upon the treatment of STS. Inhibitors of immune checkpoint proteins, such as Cytotoxic T-Lymphocyte Associated Protein 4 (CTLA-4) and Programmed cell death protein 1 (PD1), are now established standards of care across a diverse range of solid and haematological cancers that include melanoma, lung cancer, and lymphomas. The limited available data in STS indicate that the meaningful benefit of such agents is restricted to a small minority of patients that currently elude means of prospective identification. Meanwhile, engineered cellular therapies that aim to direct clonal lymphocyte responses against specific cancer-restricted antigens have showed success only in limited histological subtypes of STS.

Recent studies have challenged the notion that STS are invisible to host immunity. A significant proportion of certain STS subtypes have been shown to contain dense infiltration by immune effector cells and gene expression profiles suggestive of an active intratumoral immune response [4]. Meanwhile, associations found between poor clinical outcome and the presence of immunosuppressive factors within the STS tumour microenvironment (TME) suggest that evasion of host immunity may confer a more aggressive sarcoma phenotype [5,6,7]. Furthermore, the reported sensitivity of certain rare, genomically bland STS subtypes to immune checkpoint inhibitors (CPIs) [8] gives rise to questions that challenge the prevailing theories as to what determines a cancer’s response to such therapies. On the basis of such evidence, there remains significant hope that the beneficial reach of IO can be extended to STS.

In this review, we summarise the reported data on the efficacy of CPIs in STS, highlighting the clinical and translational findings that provide an indication of which tumours might exhibit sensitivity. We go on to explore the rationale and available evidence for the combination of CPIs with other classes of anticancer therapy in STS, as well providing an overview of the antigen-specific IO modalities under investigation in certain STS subtypes. Finally, we discuss potential avenues to broadening the effectiveness of IO within STS.

## 2. Immune Checkpoint Inhibitors in STS

**Figure 1:**
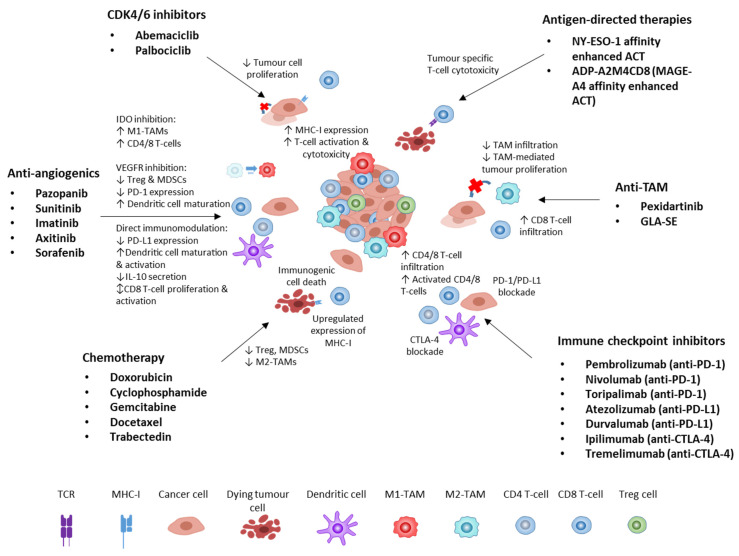
Overview of immunomodulatory agents under preclinical or clinical assessment for STS.

Cancer immunosurveillance is the now broadly accepted theory that the immune system in the body provides continual protection against the development of tumours. This is achieved through the recognition and elimination of early cancer-specific cellular aberrations that include presentation of neoantigens that result from the burden of somatic mutation within tumours [9]. Accordingly, evasion of immune-mediated elimination is now regarded as a hallmark characteristic of all cancers that develop to the point of clinical detection [10]. Cancers may employ one or more of a variety of routes to achieving immune evasion, allowing for the classification of cancers by their immune phenotype [11]. The described cancer-immune phenotypes include: (i) immune-excluded tumours, wherein the TME is shielded from immune cell infiltration by altered stromal factors, including the vascularity and constitution of the extracellular matrix; (ii) immune desert tumours, wherein the absence of pro-inflammatory factors, the presence of anti-inflammatory factors, and the avoidance of antigen presentation by tumour cells reduces the visibility of cancer to the surveillant immune system; and (iii) inflamed tumours, where infiltrates of immune effector cells within the TME exhibit evidence of an exhausted activatory state that is influenced by tumour and stromal factors as well as immunosuppressive leukocytes of both lymphoid and myeloid origin. Most T cells within a tumour show evidence of activatory exhaustion, a state associated with chronic exposure to weakly immunogenic tumour-related antigens in the absence of co-stimulatory signals and the presence of suppressive cell-cell and cytokine interactions [12]. Exhausted T cells have decreased cytotoxic potential and are characterised by high surface expression levels of inhibitory immune checkpoint receptors such as PD-1. Therapeutic monoclonal antibodies (mAb) that interrupt inhibitory immune checkpoint signalling (such as the interaction of PD-1 with its ligand PD-L1) to tumour-infiltrating lymphocytes (TILs) have been shown to successfully trigger an anti-tumour immune response (Figure 1). This response is capable of controlling and, in some circumstances, altogether eliminating tumours across a broad range of solid tumour and haematological malignancies.

### 2.1. Clinical Trials of CPIs in Mixed STS Cohorts

The earliest trials of immune checkpoint inhibitors to include STS provided little evidence of activity, with no patients responding in studies of the anti-CTLA-4 mAb ipilimumab and anti-PD1 mAb nivolumab or pembrolizumab in synovial sarcoma (SS) and uterine leiomyosarcoma (LMS), respectively (Table 1) [13,14,15]. However, subsequent larger prospective studies that recruited patients across a broader range of STS subtypes provide a strong indication of the efficacy of CPIs in a small but significant minority.

The largest series to date to treat patients with STS with immune checkpoint therapy is the SARC028 study, a US multi-institutional phase II trial of the anti-PD1 mAb pembrolizumab that aimed to establish the efficacy and safety of the drug in two cohorts consisting of 40 patients each with soft tissue or bone sarcomas [16,17]. The initial efficacy readouts reported objective tumour response in 7/40 (18%) STS patients, failing to meet a prespecified efficacy threshold of objective response rate (ORR) of 25%. However, a notable variation in pembolizumab response was seen between different STS subtypes, ranging from 4/10 (40%) in undifferentiated pleomorphic sarcoma (UPS) to 0/10 in LMS. Expansion of UPS and dedifferentiated liposarcoma (DDLPS), which are both genomically complex STS subtypes, was carried out, with a subsequent respective ORR of 9/40 (23%) and 4/39 (10%) reported in 2019 [17].

The activity of anti-PD1 monotherapy was compared to that of the combined anti-PD1/anti-CTLA-4 treatment in the ALLIANCE A091401 trial, an open-label multicentre US study that randomised patients with advanced soft tissue or bone sarcoma of various histological subtypes to receive nivolumab alone or in combination with ipilimumab [18,19]. The primary endpoint was objective radiological response, with a prespecified ORR of 13% taken to reflect evidence of clinically meaningful activity. In the primary efficacy analysis, the investigators reported a radiological response in 6/38 (16%) of the patients treated with combination nivolumab–ipilimumab. Of the six responses seen in the combination arm, three were seen to tolerate treatment beyond 60 weeks of follow-up. An expected excess of immune-related toxicities was seen with the nivolumab–ipilimumab combination, compared to nivolumab alone. Of note, of the eight responses seen across the two arms of the study, three were in patients with UPS and three with LMS. The authors concluded that, given the limited observed efficacy, nivolumab monotherapy did not warrant further investigation in unselected STS populations. However, combination nivolumab–ipilimumab showed promising activity, especially in certain STS subtypes associated with a greater degree of genomic complexity.

The results of a further series of CPIs in advanced STS have not consistently reproduced the promising efficacy signals of the SARC028 and ALLIANCE studies (Table 1). Similarly, in a retrospective assessment of patients treated at Ohio State Hospital with anti-PD1 monotherapy, only one response was seen in the 21 (5%) included patients with UPS, LMS, or DDLPS [20]. Meanwhile, the combination of anti-PD-L1 and anti-CTLA4 therapy was associated with an objective response in 1 of 16 patients with UPS, LMS, or DDLPS treated with durvalumab and tremelimumab in a prospective phase II trial performed at MD Anderson Cancer Centre [21]. More encouragingly, an overall response rate of 24% (21 of 88 patients) was reported in a US multi-institutional series of patients treated with anti-PD1/PD-L1 therapy either alone or in combination with anti-CTLA4 or other IO agents [22]. Responses were recorded in 8/25 (32%) and 9/20 (45%) of patients with UPS and LMS, respectively. Furthermore, the use of combination ipilimumab–nivolumab to treat heterogeneous STS cohorts in prospective series from China and Stanford University was associated with levels of anti-tumour activity similar to that seen in the ALLIANCE study, with responses seen in cases of UPS, LMS, and DDLPS [23,24]. Also notable are the reported efficacy results from a randomised phase II trial where patients with localised, resectable UPS or DDLPS were treated with nivolumab with or without ipilimumab for a month prior to surgery [25]. The investigators found compelling pathological evidence of significant tumour response (defined by percentage hyalinisation of the tumour) to CPIs in the resection samples of UPS (median 95% pathological response) and, to a lesser degree, DDLPS (median 22% pathological response). This pathological evidence of the CPI effect was not well reflected by radiological change in tumour size—the median change in tumour size was −4% and +9% in UPS and DDLPS, respectively, while only one partial radiological response was seen in the eight tumours that exhibited a >85% pathological response. These data suggest that, at least in the early stages of CPI treatment, the radiological appraisal of the response to immunotherapy in STS may not provide an accurate indication of the anti-tumour effect.

**Table 1:** Clinical trials and reports exploring the use of CPIs in STS.

Year Reported	Study	Intervention	Evaluable STS Patients (*n*)	ORR	Subtype Specific Objective Responses			Other Responses
					UPS (*n*)	LMS (*n*)	DDLPS (*n*)	
2013 Maki [13]	MSKCC Synovial Sarcoma pilot	Ipilimumab	6	0%	NA	NA	NA	0/6 SS
2017 Ben Ami [14]	Dana Farber uterine LMS phase II	Nivolumab	12	0%	NA	0/12	NA	
2015 Patnaik [15]	KEYNOTE-001	Pembrolizumab	1	0%	NA	0/1	NA	
2017 Groisberg [26]	MD Anderson series	CPIs in various combos	40	5%	0/1	0/12	0/6	
2017 Tawbi [16]	SARC028	Pembrolizumab	40	18%	4/10	0/10	2/10	1/10 SS
2019 Burgess [17]	SARC028 expansion cohort	Pembrolizumab	79	16%	9/40	NA	4/39	
2018 D’Angelo [18]	ALLIANCE A091401	Nivolumab	37	5%	0/5	1/15	0/3	1/1 ASPS
2018 D’Angelo [18]	ALLIANCE A091401	Nivolumab and ipilimumab	37	16%	2/6 1/6 unconfirmed	2/14	0/2	1/1 MFS 1/3 AS
2020 Chen [19]	GIST/DDLPS/UPS expansions of ALLIANCE	Nivolumab	36	6%	1/12	NA	1/12	0/9 GIST
2020 Chen [19]	GIST/DDLPS/UPS expansions of ALLIANCE	Nivolumab and ipilimumab	36	11%	2/12	NA	2/12	0/9 GIST
2020 Quiroga [20]	Ohio State series	Nivolumab or pembrolizumab	25	12%	0/3	0/5	1/6	1 inflammatory myofibroblastic sarcoma
2020 Somaiah [21]	MD Anderson phase II	Durvalumab and tremelimumab	57	14%	1/5	0/5	0/6	5/10 ASPS 1/5 Chordoma 1/5 AS
2020 Monga [22]	US multi-institute	PD1 +/− CTLA4 or other CPIs (inc. anti-CSF1R)	88	24%	8/25	9/20	NR	1 fibroblastic sarcoma 1 SEF 1 MFS
2020 Zhou [23]	Stanford series	Nivolumab and ipilimumab	38	15%	1/8 (classed as sarcoma NOS)	0/9	1/6	2/5 MFS 1/2 SFT 1/3 MPNST
2021 Chen [24]	Chinese multi-institute	Nivolumab	76	7%	NR	NR	NR	
2021 Chen [24]	Chinese multi-institute	Nivolumab and ipilimumab	74	13%	NR	NR	NR	
2020 Italiano [8]	Pooled analysis of phase II data	PD1/PDL1 +/− other CPIs	384	15%	16/103	5/82	4/61	

Abbreviations: AS, angiosarcoma; ASPS, alveolar soft part sarcoma; CPI, checkpoint inhibitor; DDLPS, dedifferentiated liposarcoma; GIST, gastrointestinal stromal tumour; LMS, leiomyosarcoma; MFS, myxofibrosarcoma; MPNST, malignant peripheral nerve-sheath tumour; NA, not applicable; NOS, not otherwise specified; NR, not recorded; ORR, objective response rate; SEF, sclerosing epithelioid fibrosarcoma; SFT, solitary fibrous tumour; SS, synovial sarcoma; STS, soft tissue sarcoma; UPS, undifferentiated pleomorphic sarcoma.

### 2.2. Immune-Based Biomarkers in Mixed STS Cohorts

The immune TME has been shown to be a source of biomarkers of prognostic and/or predictive utility across a broad range of solid cancers. The presence of more dense immune cell infiltrates within the TME has been found to be associated with a more favourable cancer outcome in multiple tumour types [27,28,29]. Similarly, gene expression signatures consistent with a more ‘immune hot’ or inflamed tumour immune phenotype have been found to predict for longer survival [30,31]. Meanwhile, the presence of higher levels of PD-L1 within the pre-treatment TME can be used to identify patient subpopulations across a range of cancer types (such as non-small cell lung cancer, bladder, and triple negative breast cancers) more likely to respond to anti-PD1 or anti-PDL1 therapy [32]. The tumour mutational burden (TMB) is reported as the number of somatic mutations per kilobase (kB) of the tumour genome and is an indirect measure of the stochastic likelihood that a mutation results in neoantigen expression within the tumour. Both the TMB and the neoantigen load have been found to predict the CPI response across a range of cancers (including colorectal and endometrial carcinoma), with high TMB (as reflected by high levels of microsatellite instability) being an established tumour-agnostic predictive biomarker for an anti-PD1 effect [33,34]. A range of further biomarkers have been identified as associated with a greater chance of a CPI response, including TME factors such as upregulation of the IFNγ-induced immune gene expression signatures [35] and host factors such as the intestinal microbiome [36] and germline HLA haplotype [37].

Compared to more common cancer types, the potential for biomarkers within the immune TME of STS is less well established. Initial studies aimed to characterise the sarcoma immune TME by immunohistochemistry (IHC)-based techniques in generally small and heterogeneous tissue cohorts. The comparability of findings between studies is limited though by variation in the IHC techniques and scoring, the immune components examined, and the clinical stage, treatment exposure, and histological type of STS included [38,39,40,41]. However, an emerging picture indicated that certain STS subtypes typically had little in the way of immune infiltrate, while other subtypes were associated with greater variety in terms of quantity and type of infiltrating immune cells, with some tumours heavily infiltrated by immune cells [42]. This was supported by the report of an international collaboration that used IHC to characterise and quantify the immune infiltrates within the TME of >1000 tumours across 22 soft tissue and bone sarcoma subtypes [4]. The key findings were that STS subtypes with single gene translocations typically had few TILs, while STS associated with greater genomic complexity showed wider variability in TIL density. No significant difference was detected in the TIL densities between non-translocation-associated STS and included reference cohorts of melanoma and various carcinomas. At least 25% of these sarcomas were found to have TIL densities greater than the median values for the reference cohorts, indicating that a non-negligible proportion of sarcomas with greater genomic complexity have TIL densities comparable to cancer types of recognised sensitivity to CPIs. The association between TILs and clinical outcome appeared to vary depending on the specific STS subtype—for example, higher levels of CD8+ T lymphocytes and CD4+ Treg cells were associated with better survival outcomes in UPS and DDLPS, whereas no association was seen in malignant peripheral nerve-sheath tumours (MPNST) between the outcome and any TIL-based characteristics. Meanwhile, very few sarcomas in this study were seen to contain tumour cell expression of PD-L1, regardless of translocation status (median 0 PD-L1-expressing tumour cells across the entire cohort). The exception to this was in AS, where the median value for the proportion of tumour cells expressing PD-L1 was around 10%. No prognostic association with PD-L1 expression was found.

More recently, a number of studies have employed multi-omic datasets derived from STS tissue cohorts for the comparative assessment of immunity-related gene expression and, through cellular deconvolution, quantitative and qualitative estimations of immune cell infiltration. In 2017, The Cancer Genome Atlas (TCGA) reported multi-omic analysis including exome and RNA sequencing from a cohort of 266 early-stage STS from across seven histological subtypes, including UPS, LMS, and DDLPS (TCGA-SARC) [43]. Among the many and varied analyses included in the original report was an assessment of the intensity and type of immune infiltrate through deconvolution of gene expression data. These findings corroborated those of the large IHC-based studies in that greater levels of immune cell infiltration were seen in genomically complex STS compared to translocation-associated STS. Furthermore, across these genomically complex subtypes was a non-trivial proportion of tumours with a high degree of infiltration by a range of lymphoid- and myeloid-derived immune cell types.

In a separate study, data from TCGA-SARC were included in an analysis of immune gene expression signatures within RNA sequencing data collected by TCGA from >10,000 tumours across 33 cancer types [30]. Unsupervised clustering of immune expression signature scores segregated the tumours into one of six immune subtypes that transcended histological classification and were associated with specific genomic and phenotypic characteristics. Within the tumours from TCGA-SARC, LMS, UPS, and DDLPS were distributed across five of the six immune subtypes, with those allocated to the C3 (inflammatory) subgroup demonstrating a trend toward a longer progression-free interval and those in C3 or C2 (interferon-gamma (IFNγ)-dominant) subgroups showing some evidence of improved overall survival (OS). A subsequent study reported in 2020 by Petitprez et al. described the de novo derivation of sarcoma immune classes (SICs) through the gene-expression-based TME deconvolution of cases of UPS, LMS, and DDLPS within four independent discovery primary STS datasets (including TCGA-SARC) [44]. Here, consensus clustering of the immune cell abundance and immune function gene signature scores identified five optimally segregated Sarcoma Immune Classes (SICs). These included a class characterised by the lowest immune cell abundance and gene signature expression (SIC A—‘immune desert); a class dominated by high expression of endothelial-cell-related genes (SIC C—‘vascularised’); and classes with heterogeneous but generally immune-low and immune-high profiles (SIC B and D, respectively). Most notable, however, was SIC E, a class characterized by the highest expression of gene signatures representing a range of immune effector cells, including those of B cell lineage. SIC E was also associated with a higher expression of a chemokine gene signature associated with the tertiary lymphoid structures (TLS), ectopic aggregations of lymphocytes, and antigen-presenting cells that are found in non-lymphoid tissues, typically at the sites of chronic inflammation that include tumours [45]. The authors went on to assess for the presence of intratumoural TLS by IHC and immunofluorescence, confirming that multiple TLS were present in a majority of tumours assigned to SIC E while almost completely absent from tumours in other SICs. Having defined and described the five SICs, the authors then assessed for prognostic associations within the pooled survival data from two of the included primary STS cohorts. They found patients with SIC A (immune desert) exhibited the shortest overall survival compared with group D or E patients (*p* = 0.048 and *p* = 0.025, respectively). In a multivariate model that included established prognostic factors such as tumour grade and patient age, the SICs were found to be significantly associated with OS in a manner independent of other factors—compared to SIC A, patients with the more immune-high SICs D and E had significantly improved survival (HR 0.373, *p* = 0.011 and HR 0.418, *p* = 0.029, respectively). When the authors then assessed for the contribution of individual cell type abundance to these prognostic associations, they found that improved OS was associated with higher levels of B cells but not with CD8+ T or cytotoxic T cells.

These IHC and gene expression studies provide compelling evidence for a subset of genomically complex STS drawn from a range of histological subtypes that exhibit an inflammatory ‘immune hot’ TME and are associated with more favourable survival outcomes. This association between immune TME suggests a biologically and clinically relevant role of host immunity in shaping the sarcoma disease phenotype. However, emerging evidence suggests that this relationship between immune TME and clinical disease behaviour may have utility beyond serving as a prognostic biomarker and may be able to prospectively identify the minority of patients who are likely to benefit from CPI therapy. So far, no association between tumour PD-L1 expression and CPI response has been observed in STS [16]. However, an analysis of matched tumour samples taken from 78 patients before and 8 weeks after commencement of pembrolizumab within the SARC028 trial found that, among the immunofluorescence-based immune TME markers assessed, higher densities of activated CD8+ T cells and PD-L1-expressing tumour-associated macrophages (TAMs) distinguished pembrolizumab responders and non-responders [46]. An association between the anti-PD1 response and higher numbers of effector T memory cells and Treg in pre-treatment samples was also observed. Meanwhile, in the study by Petitprez et al., the authors applied their SIC classifier to pre-treatment tumour samples from patients treated with pembrolizumab within the SARC028 trial [44]. Objective response to the treatment was seen in 5/10 (50%) of the patients assigned to SIC E (immune and TLS high), a rate notably higher than that seen in the other four SICs (0–25%). Similarly, PFS following pembrolizumab was longest in patients in SIC E and was significantly longer than that in SIC A (immune desert) and SIC B (heterogeneous immune-low) (*p* = 0.023 and *p* = 0.0069, respectively). Meanwhile, an expansion cohort of patients with TLS-containing tumours was treated with the combination of pembrolizumab with low-dose metronomic cyclophosphamide within the French PEMBROSARC phase II trial [47]. In the TLS-positive cohort, the ORR and 6-month PFS were 26.7% and 40%, respectively, versus 2.1% and 4.2% in all comers. The key relevance of these translational studies of patients treated with CPIs is that the distinct immune characteristics identifiable in pre-treatment tissue identify subgroups of patients enriched for response to anti-PD1 therapy. These findings require independent validation but have provided a basis for the prospective testing of immune-based biomarkers predictive of benefit from CPIs. For example, selection of patient subpopulations with TLS-containing tumours has been employed in currently recruiting trials of anti-PD1-based therapy (NCT04095208, NCT04874311, and NCT04968106).

## 3. CPI Effect in Rarer STS Subtypes

The available data from tumour-profiling studies and ‘all-comer’ clinical trials of CPIs in STS have generally indicated that both the presence of a ‘hot’ immune TME and the benefit from CPI therapy are mostly limited to a subset of tumours within genomically complex subtypes. Meanwhile, most sarcomas typified by solitary gene translocations rarely exhibit evidence of significant immune cell infiltration and have been associated with low rates of benefit from CPIs in the available trial data [4,16]. However, emerging trial evidence suggests exceptions to this, where an efficacy signal for CPIs has been seen in cohorts of patients with certain rare STS subtypes that challenge the notion that only tumours with genomic complexity and evidence of an inflamed TME will respond.

### 3.1. Alveolar Soft Part Sarcoma

Alveolar soft part sarcoma (ASPS) is a rare STS subtype with no known recognised cell of origin that exhibits a characteristic histologic appearance of nests of epithelioid tumour cells occurring within a network of connective tissue partitions containing sinusoidal blood vessels [1]. ASPS typically develops in the soft tissues of the extremities and has a high rate of metastasis to the lung, lymph nodes, and bones, with advanced disease typically following an often-indolent but ultimately fatal clinical course [48]. The pathognomonic t(X;17) translocation results in the APSCR1-TFE3 fusion gene, whose translocation product behaves as an aberrant transcription factor that drives tumorigenesis and angiogenesis [49].

A small number of patients with ASPS were included among the first heterogeneous STS cohorts to be treated with CPIs. Allowing for the small patient numbers, the response rates in ASPS stood out as among the most sensitive STS subtypes—2/4 (50%) of the ASPS patients responded to anti-PD-L1 therapy in an MD Anderson retrospective series of CPIs in the treatment of STS [26], while a response was seen in the only patient with ASPS to receive nivolumab monotherapy in the ALLIANCE A091401 trial [18]. In view of these early examples of response, further studies of CPIs with a specific focus on ASPS have been performed (Table 2). Efficacy data from an NCI-sponsored multicentre trial of the anti-PD-L1 mAb atezolizumab in ASPS was recently updated, with a report of objective response in 16/43 of the evaluable patients (37%) and a median duration of response of 16.5 months [50]. Similarly encouraging rates of response to single-agent anti-PD1 therapy were reported in a French phase II of pembrolizumab (ASPS-specific ORR 7/14 (50%)) [51,52] and two Chinese studies, where the anti-PD1 antibodies toripalimab and geptanolimab were associated with objective responses in 3/12 (25%) and 16/43 (37%) of the ASPS patients, respectively [53,54]. Meanwhile, 5/10 (50%) of the patients with ASPS responded to treatment in the MD Anderson single arm phase II trial of durvalumab and tremelimumab [21], potentially reflecting a higher degree of activity of combination anti-PD-L1/CTLA-4 therapy.

It is currently unclear what the biological properties are that sensitise a significant proportion of ASPS to CPI therapy. Dancsok et al. included eight ASPS in their IHC-based profiling of sarcoma-immune TME and found that, similar to other translocation-associated sarcomas, the TIL numbers in ASPS were scanty compared to non-translocation STS [4], suggesting that it is not the case that ASPS falls unexpectedly into an ‘immune hot’ classification. Among speculative theories as to why ASPS may respond to CPIs is the possibility that the high degree of intratumoural vascularity confers greater concentrations and/or sensitivity to the chemokines that attract immune effectors to the tumour bed or that upregulation of pro-inflammatory cytokines such as IFNγ is among the transcriptional targets of the aberrant APSCR1-TFE3 transcription factor—such theories are yet to be substantiated by translational evidence [55,56,57]. Perhaps significant is the finding of MSI-H and MMR deficiency genomic signatures in ASPS tumour samples [58,59], suggesting that ASPS may possess a significant neoantigen burden that enables subsequent antigen-specific immune attack. Alternatively, tumour-specific antigens derived from the APSCR1-TFE1 fusion protein itself could be a target for antigen-directed immune attack—the authors of an early case study of ASPS response to CPIs found in silico evidence for high-affinity stabilisation of HLA-A by at least one fusion-derived protein [60].

Regardless of the underlying mechanism of sensitivity, the available evidence provides a clear signal of the activity of anti-PD1 or anti-PD-L1 therapy in ASPS and ongoing clinical investigation of such treatments represent a highly promising therapeutic avenue for patients with this rare sarcoma subtype.

### 3.2. SWI/SNF-Deficient Sarcomas

Malignant rhabdoid tumours (MRT) are high-grade malignant neoplasms of relatively uniform rhabdoid cells characterised by the inactivation of SMARCB1, a member of the SWI/SNF complex, but otherwise harbour extremely low TMB [61]. Despite this, immunohistochemistry and scRNAseq demonstrated moderate infiltration of TILs and macrophage populations within MRT tumours, including tissue-resident, clonally expanded CD8+ T cell populations, indicating tumour-specific immunogenicity. Additionally, in vivo models showed durable responses to PD-1 blockade, and further interrogation into the mechanisms of MRT immunogenicity implicated SMARCB1 deficiency-dependent re-expression of endogenous retroviruses (ERV), inducing interferon signalling [62].

The interim results from the ongoing KEYNOTE-051 phase I-II trial have shown that one of two MRT patients included in the study demonstrated a response to pembrolizumab, while the only patient with epithelioid sarcoma, another SMARCB1-deficient tumour, also responded to treatment [63]. Rhabdoid tumours are also included in the list of responders to pembrolizumab treatment in the AcSe basket phase II study of rare sarcomas, with an objective response in 3/11 MRT patients (27%) (Table 3) [51,52]. Several case studies of checkpoint inhibitor therapy in thoracic SMARCA4-deficient sarcomas have emphasised that some patients display exceptional and durable responses to anti-PD-1 blockade, although responses were observed in both PD-L1 positive and negative SMARCA4 tumours [64,65,66]. Thus, PD-L1 expression cannot fully explain the observed sensitivity of SMARCA4-deficient tumours to anti-PD-1 blockade. Due to the significant proportion of SMARCA4-deficient STS patients who might benefit from CPIs, further translational studies in these rare STS subtypes are paramount to identify biomarkers of CPI response.

### 3.3. Clear Cell Sarcoma of Soft Tissue

Clear cell sarcoma (CCS), an aggressive soft tissue malignancy typically involving the deep soft tissues of the extremities, can be characterized by the presence of a balanced t(12;22)(q13;q12) translocation, fusing the EWS gene to the transcription factor ATF1 [67]. The EWS-ATF1 fusion protein constituently upregulates the expression of microphthalmia transcription factor (MITF), a gene implicated in the pathogenesis of melanoma; hence, this disease displays striking similarity to malignant melanoma, including the upregulation of melanocyte differentiation antigens such as HMB45 [68]. The success of immunotherapy in melanoma has, therefore, prompted trials to assess whether the same clinical benefit can be observed in CCS patients. Thus far, immune checkpoint blockade in CCS has produced durable responses in a small number of patients, as exemplified by the outcomes of 11 metastatic CCS treated with immune checkpoint inhibitors at MD Anderson (Table 3). Of these 11 patients, one had a durable response to pembrolizumab (41.8 months), although in 10/11 cases a PFS of <6 months was observed [69]. A phase II, single arm study is currently ongoing to evaluate the efficacy of TSR-042, a novel PD-1 inhibitor, in patients with advanced or metastatic CCS (NCT04274023).

**Table 3:** Clinical trials and reports exploring the use of CPIs in SWI/SNF-deficient sarcomas, clear cell sarcomas and angiosarcomas.

Year/Author	Trial	Agents	Rare Subtypes Evaluable (*n*)	ORR of Rare Subtype	Other Outcomes
2020 Geoerger [63]	KEYNOTE-051 phase I-II	Pembrolizumab	MRT (2) EPS (1)	50% MRT 100% EPS	
2021 Blay [52]	AcSé Pembrolizumab phase II	Pembrolizumab	MRT (11)	27%	18.2% 12-month PFS rate (MRT)
2019 Jones [69]	MD Anderson case series	CPI	CCS (11)	NR	1/11 Durable response (41.8 months) 10/11 <6 months response duration
2020 Painter [70]	Angiosarcoma project series	CPI	AS (6)	33%	2 CR (2/3 HNFS AG)
2020 Somaiah [21]	MD Anderson phase II	Durvalumab and tremelimumab	AS (5)	20%	1 PR (1/1 cutaneous AS)
2019 Florou [71]	Miami Miller case series	Pembrolizumab, AGEN1884, Pembrolizumab and axitinib	AS (7)	NR	71% PR rate at 12 weeks 1/2 CR to AGEN1884
2021 Wagner [72]	DART phase II	Ipilimumab and nivolumab	AS (16)	25%	60% HNFS AS ORR 2 SD 38% 6-month PFS rate

Abbreviations: AS, angiosarcoma; CCS, clear cell sarcoma; CPI, checkpoint inhibitor; CR, complete response; EPS, epithelioid sarcoma; HNFS, head, neck, face, and scalp; MRT, malignant rhabdoid tumour; NR, not recorded; ORR, objective response rate; PFS, progression-free survival; PR, partial response; SD, stable disease.

### 3.4. Angiosarcoma

In contrast to many other STS subtypes, whole exome sequencing (WES) of angiosarcomas of the head, neck, face, or scalp (HNFS) revealed a high TMB associated with ultraviolet (UV) damage mutational signature, suggesting UV-driven angiosarcomas might respond well to CPI therapies. Indeed, complete responses were observed in two of three HNFS angiosarcoma patients from this WES cohort who underwent anti-PD-1 therapy, while no clinical benefit was observed in the three non-HNFS angiosarcoma patients who underwent CPI treatment (Table 3). The HNFS patients who responded to CPI treatment displayed a high TMB, suggesting TMB as a biomarker for angiosarcoma patients who would likely benefit from CPIs [70]. Further evidence for a CPI response in a subset of AS patients is shown in the MD Anderson phase II trial, where a partial response to dual durvalumab and tremelimumab was observed in 1/5 AS patients, of which the responder was the only cutaneous AS patient in the study [21].

In a retrospective analysis of seven locally advanced or metastatic angiosarcoma patients treated with CPI therapy, the majority of which were cutaneous, a partial response rate of 71% was reported at 12 weeks. One cutaneous angiosarcoma patient even demonstrated a complete response to low-dose AGEN1884 monotherapy, an anti-CTLA-4 antibody. Surprisingly, the patient displayed a low TMB profile, although the expression of multiple novel fusion proteins as well as cancer-testis antigens were detected [71]. An ORR of 25% to dual anti-CTLA4 and anti-PD-1 blockade was reported in a phase II trial containing nine cutaneous and seven non-cutaneous metastatic or unresectable angiosarcoma patients. Of the four angiosarcoma patients who responded to dual CPI treatment, three were cutaneous (33% ORR) and only one was non-cutaneous (14% ORR), indicating that CPI therapy might benefit cutaneous angiosarcoma patients in particular [72].

## 4. CPI Combinations

### 4.1. Anti-Angiogenics

The interplay between angiogenesis and immune suppression has led to the use of anti-angiogenic therapies as a means to potentiate an immune response, further enhanced by combinations with CPIs. Vascular endothelial growth factor (VEGF), a pro-angiogenic growth factor often overexpressed in STS tumours [73], is known to mediate immunosuppression. Mechanisms of VEGF-induced immunosuppression includes the induction of Treg and myeloid-derived suppressor cells (MDSC), the inhibition of dendritic cell (DC) maturation as well as the enhanced expression of PD-1 in CD8+ T cells [74,75,76] (Figure 1). Targeting the VEGF/vascular endothelial growth factor receptor (VEGFR) axis in tumours overexpressing VEGF was able to reverse the upregulation of checkpoint proteins on T cells [76], providing evidence that anti-angiogenic tyrosine kinase inhibitors (TKIs) may represent attractive targets for STS therapy when used in combination with immune checkpoint blockade.

George et al. analysed the mechanisms of resistance in a uterine LMS patient who responded exceptionally well to anti-PD-1 monotherapy. Immunohistochemical staining, WES, RNA-seq, and neoantigen prediction showed that the only metastatic, treatment resistant tumour to arise had reduced expression of the neoantigens responsible for the initial, potent immune response but also biallelic loss of PTEN [77]. This loss of PTEN led to the upregulation of JAK/STAT signalling as well as VEGF expression, suggesting a role for this pathway in checkpoint inhibitor therapy resistance. This outcome is supported by similar observations made in anti-PD-L1/CTLA-4-resistant melanoma and warrants the investigation into combinatorial treatment with the inhibitors of the PI3K and JAK/STAT pathway or VEGFR [78]. The addition of targeted therapies to checkpoint inhibitor regimens promises to increase the number of durable responses seen by addressing potential immune resistance mechanisms.

Immunohistochemical analysis of solitary fibrous tumour (SFT) patients treated with sunitinib revealed an increase in CD68+ CD14+ M1-like macrophages as well as CD4+ and CD8+ T cells and, upon the isolation of such cells from dissociated patient tissue, they were shown to be functionally active [79]. A similar observation was made in fibrosarcomatous dermatofibrosarcoma protuberans (DFSP) patients post-imatinib treatment, also showing an increased infiltration of functionally active T cells and M1-like macrophages in areas of pathological response [80]. Imatinib causes this immunomodulation by inhibiting expression of the immunosuppressive enzyme indoleamine 2,3-dioxygenase (IDO) in tumour cells [81].

In addition to inhibiting the immune-evasion pathways employed by tumour cells, several anti-angiogenic inhibitors currently in use or under assessment for STS treatment can also have direct, immunomodulatory effects on the immune landscape. For example, sunitinib and sorafenib, two multi-target, anti-angiogenesis TKIs reduced T cell proliferation in vitro, inducing apoptosis and leading to a reduction in CD8+ effector T cell populations. Alternatively, peripheral blood mononuclear cells (PBMCs) treated with axitinib did not lead to a reduction in CD8+ T cell subsets [82]. Pazopanib induces dendritic cell maturation and activation in vitro by inhibiting the Erk/β-catenin pathway and simultaneously reduces the production of immunosuppressive cytokine IL10 and the expression of PD-L1 leading to increased T cell activity [83]. Clearly, anti-angiogenic drugs are able to enhance immune responses, although some cause detrimental effects on effector immune populations. Thus, anti-angiogenics should be carefully selected for combinatorial trials with immunotherapy in order to maximise synergy.

The central role of angiogenesis in the development of ASPS provided a basis for the successful development of small molecule inhibitors of VEGFR (among other molecular targets) in this disease. This is best exemplified by the CASPS study, an international, placebo-controlled, randomised phase II trial of cediranib in advanced ASPS [84]. In 44 evaluable patients to undergo randomisation, the median change in target lesion at 24 weeks of treatment was −8.3% with cediranib, compared to +13.4% with the placebo, with an ORR of 19% vs. 0%.

Given the activity of anti-PD1 or anti-PD-L1 mAbs and anti-VEGFR TKIs as monotherapy in ASPS and the hypothetical scope for therapeutic synergies, there is a firm rationale for the combination of the two drug classes. The combination of the potent VEGFR inhibitor axitinib with pembrolizumab has been assessed in a single-centre phase II trial that recruited across a range of STS types, with an objective response seen in 6/11 (55%) ASPS patients (Table 4) [85]. Meanwhile, the multi-targeted TKI sunitinib has been investigated in combination with nivolumab in the ImmunoSARC trial, a multicentre phase II trial still recruiting in Europe across a range of STS subtypes. A safety report from this trial also included some early efficacy data, including details of objective response in 4/7 (57%) of the ASPS patients treated so far [86]. These response rates notably exceed those seen with TKI monotherapy in the CASPS trial—whether this is a purely additive effect of anti-PD1 and TKI therapy, as opposed to treatment synergy, is yet to be conclusively demonstrated. Additionally, the anti-PD-1 monoclonal antibody, SHR-1210 is currently undergoing phase II assessment in combination with an anti-angiogenic TKI, apatinib, in STS patients (NCT04239443).

### 4.2. Chemotherapy and Radiotherapy

Systemic chemotherapies, including anthracyclines and alkylating agents commonly used for STS treatment, are also able to modulate the tumour immune landscape to promote an anti-tumour response (Figure 1). Anthracyclines such as doxorubicin induce immunogenic cell death, stimulating a caspase-dependent anti-cancer immune response [87], while even low-dose cyclophosphamide can selectively suppress inhibitory immune subsets including, Treg cells and MDSCs, inducing IFN-γ-mediated immunity [88]. Gemcitabine or docetaxel treatment can selectively deplete MDSCs [89,90] and gemcitabine further upregulates the expression of MHC-I on tumour cells and subsequent tumour-antigen cross-presentation [91].

Additionally, cytotoxicity against TAMs has been shown to be a key component of trabectedin’s antitumor activity when used to treat sarcoma-bearing mice, while levels of circulating and tumour-infiltrating macrophages have been shown to reduce with trabectedin treatment in patients with LMS [92]. In an immunocompetent fibrosarcoma mouse model, trabectedin treatment led to an intratumoural upregulation of T-cell-associated markers, although PD-1 expression was also increased. While this fibrosarcoma model displayed a poor response to anti-PD-1 treatment, trabectedin pre-treatment was able to induce sensitivity to anti-PD-1 therapy [93]. Additionally, 3D, in vitro, and zebrafish models of UPS, liposarcoma (LPS), and LMS have shown that the extracellular matrix also plays a role in the trabectedin response via timp1 upregulation [94]. The potential for an immunomodulatory component of trabectedin’s activity in LMS has informed currently recruiting studies investigating the combination of trabectedin with immune checkpoint therapy (NCT03085225, NCT03138161, and NCT03590210).

**Table 4:** Combinatorial CPI clinical trials in STS.

Year/Author	Trial	Agents	Evaluable Patients (*n*)	ORR	Other Outcomes
2019 Wilky [85]	Miami single-centre phase II	Pembrolizumab and axitinib	30	27% (55% ASPS ORR)	8 PR (6 ASPS, 1 ES, 1 LMS) 9 SD 3 minor responses (1 LMS, 1 SS, 1 UPS) 4.7-month mPFS
2020 Martin-Broto [86]	IMMUNOSARC multicentre phase Ib/II	Nivolumab and sunitinib	58 (12 phase Ib, 46 phase II)	21% (57% ASPS ORR)	Phase II: 1 CR 5 PR 33 SD 7 PD
2017 Toulmonde [93]	PEMBROSARC phase II	Pembrolizumab + metronomic cyclophosphamide	50	2%	1 PR 16 SD 31 PD 2 minor responses
2021 Italiano [47]	Tertiary lymphoid structure selected PEMBROSARC phase II	Pembrolizumab + metronomic cyclophosphamide	30	27%	5 SD 4.1-month mPFS
2017 Weiss [95]	PembroPlus phase 1b	Pembrolizumab and doxorubicin/gemcitabine/docetaxel	6	0%	1 SD 5 PD
2020 Pollack [96]	Phase I/II	Pembrolizumab and doxorubicin	37	19%	7 PR 2 unconfirmed PR 11 SD 8.1-month mPFS

Abbreviations: ASPS, alveolar soft part sarcoma; CR, complete response; ES, epithelioid sarcoma; LMS, leiomyosarcoma; mPFS, median progression-free survival; ORR, objective response rate; PD, progressive disease; PR, partial response; SD, stable disease; SS, synovial sarcoma; UPS, undifferentiated pleomorphic sarcoma.

The PEMBROSARC trial combining pembrolizumab and metronomic cyclophosphamide demonstrated limited activity in a cohort of advanced LMS and UPS patients (Table 4), with no patients free of progression at 6 months. In other STS subtypes, the 6-month progression free survival rate was 11.1% with one partial response. Translational studies of the patient tumours revealed high macrophage counts, and these cells expressed the immunosuppressive enzyme IDO. Additionally, archival tumour tissue from the only responding patient showed the highest number of immune cells expressing PD-L1 of the trial cohort [95]. The immunosuppressive tumour microenvironment and the lack of PD-L1-expressing immune cells is therefore implicated in the primary resistance of STS tumours to CPIs and chemotherapy treatment. To address these potential resistance mechanisms, PEMBROSARC is currently assessing the addition of an anti-IDO1, TLR4 agonist or EZH2 inhibitor to the combined pembrolizumab and metronomic cyclophosphamide treatment in advanced STS patients (NCT02406781) [96].

Other clinical trials combining chemotherapy with CPIs have also displayed limited response in STS patients. Zero out of seven responses were observed in the small STS cohort of PembroPlus, combining pembrolizumab with multiple different chemotherapies used in standard-of-care STS treatment, including doxorubicin, gemcitabine, and docetaxel [97]. A phase I/II study of pembrolizumab and doxorubicin treatment in anthracycline-naïve, advanced STS patients achieved an ORR of 19%, which did not meet the prespecified ORR, although the trial reported a disease control rate of 81%. Additionally, the median PFS was markedly improved in patients receiving combinatorial treatment compared to doxorubicin monotherapy (8.1 and 4.1 months, respectively) [98]. Combination CPI treatment with gemcitabine is under phase I assessment in UPS and LMS patients (NCT03123276), while combinatorial treatment with pembrolizumab and eribulin is under phase II clinical assessment in LPS, LMS, and UPS patients (NCT03899805).

The treatment of high-risk-of-recurrence STS patients with CPI and concurrent radiotherapy is another, possibly synergistic, combination of interest, with the hypothesis that radiotherapy can aid responses by abscopal effects and simultaneously induce anti-tumour immunity. A case report of a recurrent thoracic CCS patient showed a complete response when treated with pembrolizumab and radiotherapy [99]. Following this observation, a large phase II trial will assess the efficacy of neoadjuvant radiotherapy and pembrolizumab prior to surgical resection with adjuvant pembrolizumab treatment versus neoadjuvant radiotherapy and surgical resection alone in a broad range of STS subtypes (NCT03092323). Meanwhile, several smaller trials are utilising dual CPI treatments, such as ipilimumab and nivolumab (NCT03463408) and durvalumab and tremelimumab (NCT03116529), instead of single-agent checkpoint inhibitor treatment.

### 4.3. CDK4/6 Inhibitors

The inhibition of cyclin-dependent kinases 4 and 6 (CDK4/6) has emerged as a potential treatment option for a select number of STS subtypes, such as SS, due to the potent cell cycle arrest and induction of apoptosis observed in vitro [100]. In other cancer types the CDK4/6 inhibitor responses have been shown to be partially mediated by the induction of an immune response and can also act as direct immune modulatory agents [101,102,103]. In vitro T cells treated with CDK4/6 inhibitor abemaciclib only demonstrated a modest, transient reduction in proliferation at clinically relevant concentrations but upregulated the genes associated with T cell activation, including IL-2 and TNF [104]. The abemaciclib response in murine models of hormone receptor-positive breast cancer was associated with an intratumoural T cell inflammatory immune signature, enhancing antigen presentation. The synergistic combination of CDK4/6 inhibition with PD-1L checkpoint blockade therapy led to complete regression and immunological memory [104]. Similar results have been shown in SS cell lines, where treatment with abemaciclib in vitro induced MHC-I surface expression and T cell activation when cultured with pre-treated SS cells, leading to enhanced NY-ESO-1-directed T cell cytotoxicity (Figure 1) [105].

LPS could also benefit greatly from a combination of CDK4/6 inhibition and immunotherapy due to the upregulation of CDK4 observed in the majority of well-differentiated liposarcoma (WDLPS) and DDLPS. The mechanistic synergy demonstrated in other tumour types plus the promising clinical trial results of LPS patients treated with CDK4/6 inhibitor monotherapy [106] has now provided the rationale for the phase II clinical evaluation of the CDK4/6 inhibitor palbociclib combined with PD-1 blockade (INCMGA00012) in patients with advanced WD/DDLPS (NCT04438824).

## 5. Anti-Tumour-Associated Macrophage Approaches

Macrophages are myeloid-derived cells of the innate immune system that play a number of crucial roles in mediating adaptive immune responses, including antigen presentation and influencing immune state through paracrine and endocrine mechanisms. The biological and clinical relevance of TAMs remains to be definitively characterised in many cancer types, reflecting the complex and dynamic nature of macrophage function. It is recognised that macrophages demonstrate functional plasticity within the tumour micro-environment and, depending on an immune state that may be influenced by a range of tumour and host factors, may differentiate into either anti-tumorigenic, pro-inflammatory M1, or pro-tumorigenic anti-inflammatory M2 functional states [107,108].

The biological understanding of TAMs underpinning sarcoma progression is largely unknown but has experienced a surge of interest in recent years with several preclinical studies reported. Shiraishi et al. demonstrated that in co-culture, CD163+ macrophages, a marker of the M2 functional state, stimulated the proliferation of LMS and myxofibrosarcoma cell lines, and this macrophage-induced tumour proliferation was reduced upon CD163 silencing. They observed that in murine CD163-deficient macrophages the expression of IL6 was decreased, while the silencing of IL6 expression in wildtype macrophages was sufficient to negate the macrophage-induced proliferation of murine fibrosarcoma cells [109]. Further evidence of the pro-tumorigenic cross-talk between sarcoma cells and TAMs was demonstrated in uterine LMS cells, where overexpression of maternal embryonic leucine zipper kinase (MELK), a serine/threonine kinase involved in cell cycle, apoptosis, and splicing regulation, contributed to doxorubicin chemoresistance both through an autonomous JAK2/STAT3 anti-apoptotic mechanism and through M2 macrophage polarisation via miR-34a/JAK2/STAT3. This study suggests a combination of doxorubicin and MELK inhibitor treatment could act synergistically by preventing M2 TAM polarisation and thus increasing doxorubicin sensitivity in uterine LMS [110], although MELK inhibition has yet to be assessed in STS in a clinical setting.

Pre-clinical studies of anti-TAM agents in other subtypes have shown that pexidartinib, an inhibitor of colony stimulating factor receptor-1 (CSF-1R), depletes tumour-infiltrating macrophages and suppresses tumour growth in MPNST xenografts [111], while suppressing tumour growth and increasing the infiltration of CD8+ T cells in an orthotopic osteosarcoma model [112]. Targeting the tumorigenic potential of macrophages is particularly relevant in tenosynovial giant cell tumours (TGCT), which overexpress colony stimulating factor 1 (CSF-1), leading to the recruitment of CSF-1R+ macrophages [113] (Figure 1). Treatment with pexidartinib indeed generated a robust tumour response in TGCT patients [114], leading to recent FDA approval for inoperable TGCT. Following this success, the combination of pexidartinib with the mTOR inhibitor sirolimus is under phase II assessment in advanced MPNST and phase I assessment in advanced, non-MPNST STS patients (NCT02584647). A further anti-TAM therapy under phase I clinical investigation in metastatic or inoperable STS is GLA-SE, which is an agonist of Toll-like receptor 4 (TLR4), an activating receptor expressed by innate immune cells, including macrophages (NCT02180698).

## 6. Antigen-Directed Therapies

Current understanding reflects that responses to immune checkpoint inhibitor therapy are mediated by the cytotoxic T cell recognition of largely non-recurrent tumour antigens that result stochastically from somatic mutation. In contrast, certain STS subtypes have been shown to frequently express recurrent tumour-specific proteins that could provide the basis for antigen-directed immune responses. Cancer-testis antigens (CTA) are a group of over 40 identified proteins whose physiological expression is limited to embryological tissues but whose aberrant expression has been demonstrated in a range of cancers. NY-ESO-1 is a CTA that has been shown to be expressed in a large proportion of SS and myxoid/round cell (high-grade myxoid) liposarcoma (MRCLS) and has been the focus of research that aims to generate antigen-directed anti-tumour immunity [115,116].

The production of clinical grade, autologous NY-ESO-1-specific T cells has been shown to be feasible through the ex vivo selection and expansion of antigen-specific populations from PBMC isolated from sarcoma patients [117]. Alternatively, autologous T cells can be genetically modified to express TCR with enhanced affinity binding to MHC-bound, NY-ESO-1-derived antigens, such as NY-ESO-1^c259^ T cells (Figure 1). The use of the latter approach for adoptive T cell therapy (ACT) is under clinical investigation in pilot phase I studies, recruiting patients with MRCLS or SS (Table 5). Preliminary results of these trials show tumour shrinkage, resulting in 2/2 unconfirmed partial responses in MRCLS and an ORR of 50% with one complete response in SS [118,119]. Affinity-enhanced ACT with adjuvant IL-2 achieved an objective tumour response rate of 61% in the treatment of a small, early phase trial cohort of patients with NY-ESO-1-expressing SS following lymphodepletion by cyclophosphamide and fludarabine [120]. A multi-cohort phase I/II trial further studied the biomarkers associated with gene-modified, NY-ESO-1-directed ACT response and identified that T cell expansion shortly after infusion was associated with tumour response, and this also was associated with high NY-ESO-1 expression. Post infusion biopsies showed no significant change in total CD3+ CD8+ T cells despite a slight increase and a leukocyte infiltration and did not correlate with a response. While antigen loss can be attributed to resistance to T-cell-directed therapies, NY-ESO-1 expression appeared to be unaffected by gene-modified T cell infusion in all four cohorts studied [121].

Alternative CTAs such as MAGE-A4 have demonstrated expression in the majority of SS tumours (82.2%) as well as MRCLS (67.7%) [122]. Preliminary results from the SURPASS phase I trial, utilising ADP-A2M4CD8, an autologous, affinity-enhanced T cell therapy targeting MAGE-A4 and also expressing CD8α co-receptor, supports the use of these antigen-directed T cells in SS and MRCLS (NCT04044859). Recruitment has since begun for the phase II SPEARHEAD1 trial (NCT04044768) after the FDA designated ADP-A2M4CD8 as a regenerative medicine advanced therapy. SS tumours often display immune-suppressed microenvironments and are largely unresponsive to immune checkpoint inhibitors. Therefore, antigen-directed therapies represent a much-needed alternative approach to immunotherapy in this particular subtype and could perhaps show efficacy in other subtypes which have displayed similar disappointing responses to immune checkpoint inhibitors. Although previous, affinity-enhanced ACT directed towards antigens such as MAGE-A3 have shown serious adverse events due to the engineered TCR displaying off-target effects [123], recent NY-ESO-1 and MAGE-A4 affinity-enhanced ACT variants have shown acceptable toxicity profiles [121,122]. However, wider clinical adoption will be challenging in face of the high technical demands and expense of such therapies.

## 7. Future Directions

### 7.1. Immunomodulation Approaches

#### 7.1.1. Immunogenic Cell Death

Immunogenic cell death is a form of cell death, which releases damage-associated molecular patterns (DAMPs). Dendritic cells can present DAMPs derived from dying tumour cells, which initiates anti-tumour T cell immunity. Activation of receptor-interacting serine/threonine protein kinase 1 (RIPK1) as well as formation of ripoptosome complexes are important components for immunogenic cell death and can be stimulated by TNF to enhance antigen cross priming of CD8+ T cells [124]. However, the inhibitors of apoptosis (IAP) are frequently overexpressed in tumour cells, protecting them from TNF-induced, RIPK1-mediated cell death [125]. A recent study aimed to potentiate the immunogenic activity of RIPK1-mediated cell death in a rat LPS model by employing isolated limb perfusion (ILP) with TNF and melphalan treatment, a standard-of-care treatment for inoperable STS of the extremities [126]. Adding a second mitochondria-derived activator of caspase (SMAC) mimetic to this protocol in order to target IAP sensitised the LPS models to RIPK1-induced cell death, delaying local recurrence and activating intratumoural CD8+ T cells and NK cells. The addition of immune checkpoint inhibitors following this ILP treatment regimen further delayed local recurrence [126]. The use of SMAC mimetics to potentiate immunogenic cell death and enhance immunotherapy response is an attractive avenue for STS treatment. Other standard-of-care therapies should therefore be analysed to assess the extent of RIPK1-mediated cell death caused in order to highlight synergistic combinations.

#### 7.1.2. LXR Agonists

Another potential avenue for STS immunotherapy is the therapeutic depletion of MDSCs. These cells demonstrate elevated infiltration and expansion in several cancer types, suppressing innate and adaptive immune responses against cancer cells via the secretion of immune-suppressive cytokines [127]. Activating liver-X nuclear receptor (LXR) reduces MDSC viability via the action of transcriptional target gene apolipoprotein E (ApoE). Therapeutic agonism of LXR reduced the intratumoural and circulating MDSC levels in murine models of melanoma, glioblastoma, lung cancer and ovarian cancer, reversing tumour immune evasion [128]. The LXR agonist RGX-104 is now in a phase I clinical trial (NCT02922764) of which initial results show that RGX-104 treatment induced a reduction in granulocytic MDSCs in patients of multiple cancer types, including sarcoma [128]. The results of such trials are eagerly awaited, though further studies with multiple STS subtypes should be conducted to identify potential subtype specific responses.

#### 7.1.3. EZH2 Inhibitors

Clinical trial studies are now emerging based on the therapeutic inhibition of the histone methyltransferase EZH2, a key epigenetic regulator found to suppress the immunogenicity of tumour cells via transcriptional repression of genes involved in IFNγ response and antigen presentation [129]. However, EZH2 also functions as an essential regulator of immune cell development and inhibition can have a negative or positive impact on T cell development, depending on the stage of immune response [130]. In addition, the loss of PRC2 function, a complex containing EZH2 as the catalytic component, is important to MPNST tumourigenesis via the transcriptional activation of PRC2-repressed homobox master regulators [131]. However, loss of function mutations are observed in other components of PRC2 in MPNST patients and not in EZH2, while, paradoxically, some MPNST overexpress EZH2, suggesting that EZH2 may still have an oncogenic function outside of PRC2 via non-canonical pathways [132]. EZH2 is essential for Treg lineage commitment and has been shown to suppress NK mediated antitumour immunity [130], providing the rationale for clinical assessment of EZH2 inhibition. Planned clinical trials include a phase II study, utilising the EZH2 inhibitor tazemetostat in combination with immune checkpoint inhibitor durvalumab and will include a cohort of STS patients (NCT04705818).

#### 7.1.4. BO-112

BO-112 is a non-coding double stranded RNA with a polyethylamine carrier, formulated to improve intracellular delivery, which acts as an agonist to Toll-like receptor 3 (TLR3), retinoic acid-inducible gene I (RIG-1), and melanoma differentiation-associated gene 5 (MDA-5). This therapy was shown in preclinical studies to cause immunogenic cell death whilst engaging innate and adaptive immunity towards tumours, thus increasing the effectiveness of combined immune-checkpoint inhibition [133]. The results from a first-in-human clinical trial (NCT02828098) combining B0-112 with anti-PD-1 therapy demonstrated tumour cell necrosis, apoptosis, and immune re-activity, suggesting this therapy could be used to overcome immune-checkpoint inhibitor-resistant tumours [134]. Another clinical trial is currently assessing the use of B0-112 via intratumoural delivery along with nivolumab treatment for resectable STS (NCT04420975).

#### 7.1.5. Oncolytic Virus

Another treatment methodology aiming to potentiate an anti-tumour immune response is the use of oncolytic viruses such as Talimogene laherparepvec (T-VEC). T-VEC is a modified version of the herpes simplex virus type-1, designed to selectively replicate within and lyse tumour cells, stimulating a local and systemic anti-tumour immune response due to the release of tumour-specific antigens [135]. Further modifications of T-VEC include the expression of granulocyte macrophage colony-stimulating factor (GM-CSF) to promote the activation of antigen-presenting cells and deletion of the ICP47 gene to prevent viral-mediated suppression of antigen presentation [135].

The effectiveness of combined intratumour T-VEC and intravenous pembrolizumab treatment in advanced STS patients was assessed in a phase II clinical trial, demonstrating induced anti-tumour activity across a range of subtypes with a manageable safety profile and an ORR of 35% [136]. Furthermore, an ongoing phase II study will assess the efficacy of JX-594 (Pexa-Vac), a targeted oncolytic vaccinia virus [137], in advanced STS patients combined in one arm with metronomic cyclophosphamide alone and in another arm with metronomic cyclophosphamide and avelumab (NCT02630368).

### 7.2. Targeting Alternative Immune Checkpoints

Beyond both the PD-1 and CTLA-4 axis, a number of additional cell surface receptor-ligand interactions are known to contribute to the complex regulation of T cell functional activation and have been implicated in modulating the tumour immune microenvironment toward either pro-inflammatory and anti-tumour or immunosuppressive and tumour-promoting phenotypes. Additional checkpoint inhibitor proteins whose expression is progressively increased during T cell exhaustion include lymphocyte activation gene 3 protein (LAG3, also known as CD223) and T cell immunoglobulin and mucin domain-containing 3 (TIM3, also known as HAVCR2) [138]. Both of these proteins have been shown to be expressed on tumour-infiltrating regulatory CD4 + FOXP3+ T cells (Treg) and Th1 CD4+ T cells, respectively, suggesting a potential role in immune escape and/or evasion [139,140]. V-set domain-containing T cell activation inhibitor 1 (VTCN1, also known as B7.H4) is expressed on antigen-presentation cells and on ligand binding and inhibits pro-inflammatory Th1-type activation of T cells [141]. High levels of tumour cell expression of LAG3, TIM3, and VTCN1 have been reported in multiple cancer types, including sarcoma, often in association with a worse clinical outcome, indicating an active role in promoting an immunosuppressive, tumour-promoting microenvironment [4,140,142,143]. Inhibitory therapeutic mAbs against these checkpoint proteins are in development with anti-LAG3 mAbs under active clinical investigation combined with other checkpoint inhibitors (NCT03964233 and NCT04095208).

## 8. Conclusions

Historically, the progress of IO in sarcoma has been lacking when compared to other cancer types, such as melanoma and NSCLC, primarily due to the rare and heterogeneous nature of this group of diseases. While patient outcome remains poor, recent evidence from both pre-clinical and clinical studies provides hope for an alternative therapeutic strategy based on modulation of the immune system. The development of immune-based biomarkers that may serve to inform clinical decision making regarding the use of immunotherapy and other treatment modalities will depend upon further analysis of existing retrospective and new prospective studies. Additional pre-clinical work and better in vitro and in vivo models are also necessary in order to characterise distinct resistance mechanisms and hence propose salvage or combination treatment regimens to address this. Toxicities related to immune-related adverse events (irAEs) also influence the choice of IO to use in individual patients. Despite these challenges, great progress has been made within the field of STS IO with increased translational research and innovative therapeutics, which holds an incredible potential to address the unmet needs of this patient group in the coming years.

## Figures and Tables

**Table 2:** Clinical trials exploring the use of CPIs in ASPS.

Year/Author	Trial	Agents	Evaluable ASPS Patients (*n*)	ORR	Other Outcomes
2017 Groisberg [26]	MD Anderson series	Anti-PD1	4	50%	2 PR 2 SD
2018 D’Angelo [18]	ALLIANCE A091401	Nivolumab	1	100% (PR)	
2018 D’Angelo [18]	ALLIANCE A091401	Nivolumab and ipilimumab	1	0%	
2021 Naqash [50]	NCI single arm phase II	Atezolizumab	43	37%	1 CR 14 PR 1 unconfirmed PR 25 SD 16.5-month mDOR
2021 Blay [52]	AcSe single arm phase II French multicentre	Pembrolizumab	14	50%	7.5-month mPFS 36% 12-month PFS rate
2020 Yang [53]	Beijing phase I	Toripalimab	12	25%	1 CR 4 PR 11.1-month mPFS
2020 Shi [54]	Gxplore-005 Multicentre Chinese single arm phase II	Geptanolimab	37	38%	14 PR 6.9-month mPFS 3-month PFS rate 70% 6-month PFS rate 56%
2020 Somaiah [21]	MD Anderson phase II	Durvalumab and tremelimumab	10	50% (PR)	90% 3-month PFS rate
2020 Italiano [8]	Pooled analysis of phase II data	PD1/PDL1 ± other CPIs	41	49%	

Abbreviations: ASPS, alveolar soft part sarcoma; CPI, checkpoint inhibitor; CR, complete response; mDOR, median duration of response; mPFS, median progression free survival; ORR, objective response rate; PFS, progression-free survival; PR, partial response; SD, stable disease.

**Table 5:** Clinical trials exploring the use of ACT in STS.

Year/Author	Trial	Agents	Evaluable Patients (*n*)	ORR	Other Outcomes
2018 D’Angelo [118]	Phase I/II	NY-ESO-1^c259^ T cells	MRCLS (2)	100% unconfirmed PR	
2018 D’Angelo [119]	Phase I/II	NY-ESO-1^c259^ T cells	SS (12)	50%	1 CR 5 PR
2015 Robbins [120]	Phase I/II	NY-ESO-1 affinity-enhanced T cells Adjuvant IL-2	SS (18)	61%	2 CR
2019 Ramachandran [121]	Phase I/II cohort expansion	NY-ESO-1^c259^ T cells	SS (42)	Cohort 1: 50% Cohort 2: 40% Cohort 3: 20% Cohort 4: 27%	1 CR 14 PR 24 SD 3 PD

Abbreviations: CR, complete response; IL-2, interleukin-2; MRCLS, myxoid/round cell liposarcoma; NY-ESO-1, New York esophageal squamous cell carcinoma 1; ORR, objective response rate; PR, partial response; PD, progressive disease; SD, stable disease; SS, synovial sarcoma.

## Data Availability

Data sharing not applicable.
